# Ginger Constituent 6-Shogaol Attenuates Vincristine-Induced Activation of Mouse Gastroesophageal Vagal Afferent C-Fibers

**DOI:** 10.3390/molecules27217465

**Published:** 2022-11-02

**Authors:** Mayur J. Patil, Yongming Huang, Mingwei Yu, Xinzhong Dong, Bradley J. Undem, Shaoyong Yu

**Affiliations:** 1 Department of Medicine, Johns Hopkins University School of Medicine, Baltimore, MD 21205, USA; 2 Solomon H. Snyder Department of Neuroscience, Johns Hopkins University School of Medicine, Baltimore, MD 21205, USA

**Keywords:** emesis, vincristine, TRPA1, 6-shogaol, vagal, nodose, gastrointestinal tract

## Abstract

Chemotherapeutic agent-induced nausea and vomiting are the severe adverse effects that are induced by their stimulations on the peripheral and/or central emetic nerve pathways. Even though ginger has been widely used as an herbal medicine to treat emesis, mechanisms underlying its neuronal actions are still less clear. The present study aimed to determine the chemotherapeutic agent vincristine-induced effect on gastroesophageal vagal afferent nerve endings and the potential inhibitory role of ginger constituent 6-shogaol on such response. Two-photon neuron imaging studies were performed in ex vivo gastroesophageal-vagal preparations from Pirt-GCaMP6 transgenic mice. Vincristine was applied to the gastroesophageal vagal afferent nerve endings, and the evoked calcium influxes in their intact nodose ganglion neuron somas were recorded. The responsive nodose neuron population was first characterized, and the inhibitory effects of 5-HT3 antagonist palonosetron, TRPA1 antagonist HC-030031, and ginger constituent 6-shogaol were then determined. Vincristine application at gastroesophageal vagal afferent nerve endings elicited intensive calcium influxes in a sub-population of vagal ganglion neurons. These neurons were characterized by their positive responses to P2X_2/3_ receptor agonist α,β-methylene ATP and TRPA1 agonist cinnamaldehyde, suggesting their nociceptive placodal nodose C-fiber neuron lineages. Pretreatment with TRPA1 selective blocker HC-030031 inhibited vincristine-induced calcium influxes in gastroesophageal nodose C-fiber neurons, indicating that TRPA1 played a functional role in mediating vincristine-induced activation response. Such inhibitory effect was comparable to that from 5-HT3 receptor antagonist palonosetron. Alternatively, pretreatment with ginger constituent 6-shogaol significantly attenuated vincristine-induced activation response. The present study provides new evidence that chemotherapeutic agent vincristine directly activates vagal nodose nociceptive C-fiber neurons at their peripheral nerve endings in the upper gastrointestinal tract. This activation response requires both TRPA1 and 5-HT3 receptors and can be attenuated by ginger constituent 6-shogaol.

## 1. Introduction

Nausea and vomiting are adverse effects that are commonly elicited by cancer chemotherapy agents. These adverse events not only add extra suffering to the patient, but may also lead to discontinuation of the treatment [1]. There are few therapeutic options available to relieve these symptoms, due mainly to our incomplete understanding of the neuronal mechanism underlying chemotherapeutic agent-induced emetic reflex. Vagal afferent nerve fibers are richly distributed in the gastrointestinal (GI) tract, where they can process different types of noxious stimuli, such as mechanical distension, ingested toxins, and chemical irritants, that might trigger emetic reflex [2,3]. The transduction of noxious stimuli into action potential discharges depends on specific ion channels/receptors that are selectively expressed at vagal afferent peripheral nerve endings. The elicited action potential discharges are then conducted to the central nervous system and subsequently evoke emetic reflex in order to release mechanical tension or expel toxic agents or organisms. Even though vagal afferent nociceptive C-fibers have recently been characterized to play crucial roles in processing such noxious stimuli [4,5,6], the specific afferent nerve subtype that encodes chemotherapy agent-elicited actions in the GI tract remains ill-defined.

Ginger (*Zingiber officinale*) has a long history and worldwide application as an herbal medicine for certain medical conditions. One of the major complementary applications of ginger is to treat nausea and vomiting [7]. Recent clinical studies have demonstrated that ginger could effectively relieve nausea and vomiting in pregnant women and post-surgical patients [8]. In animal models, ginger has been shown to decrease emetic episodes in dogs challenged by cisplatin [9]. Previous studies indicated that ginger extracts might exert antiemetic effects at the level of the gastrointestinal system [7], but the neuronal mechanism of this action is still unclear.

Ginger is a multi-faceted remedy. Clinical application of different ginger remedies (ginger powder, fresh ginger, dry ginger, and single ginger extract) often leads to controversial results [8]. It is now clear that pharmacological functions of ginger are mainly produced by its bioactive phytochemicals, including the pungent principle gingerols (including 4-, 6-, 8, 10-, and 12-gingerols) that are mainly in fresh ginger and their dehydrated product shogaols (including 6-, 8-, and 10-shogoals) in dried ginger [10]. Dehydrated from 6-gingerol under heat or acidic conditions, 6-shogaol is more potent and effective in its bioactivities. The 6-shogaol constituent can regulate gastrointestinal sensory motor functions via its activities on cholinergic M receptor and serotonergic 5-HT receptors, which might impact emetic reflex and symptoms [11]. The 6-shogaol constituent has been shown to non-competitively inhibit 5-HT-evoked responses in a dose-dependent manner in vagal nodose neurons, which might contribute to its antiemetic effect [12]. Our recent study demonstrated that 6-shogaol not only activated but also desensitized gastroesophageal vagal nociceptive C-fiber neurons. TRPA1 played an important role in mediating such effects [13].

Almost all chemotherapy agents, despite their disparate chemical structures, can induce nausea and vomiting, suggesting a generalized neuronal action [14]. Among them, vincristine is known for its autonomic and gastrointestinal side effects [15,16]. In the present study, we aimed to determine whether vincristine directly activates vagal afferent nerve terminals in the upper GI tract. We also evaluated the potential inhibitory role of ginger constituent 6-shogaol on such response. We applied our newly established two-photon neuron imaging technique in ex vivo gastroesophageal-vagal preparation from Pirt-GCaMP6 mice. This approach allows us to more efficiently characterize relatively large numbers of responsive vagal afferent fibers simultaneously in a single experiment.

## 2. Results

### 2.1. Vincristine Directly Activated Vagal Afferent Neurons at Their Gastroesophageal Nerve Terminals

To determine whether vincristine directly activated gastroesophageal nodose afferent nerve fibers, we performed two-photon imaging studies in ex vivo gastroesophageal-vagal preparations from Pirt-Cre;R26-GCaMP6s mice. One of the major advantages of two-photon imaging is that it allows us to study simultaneously about 150 nodose neurons per preparation. It also enables us to track the responsiveness of each fiber to multiple stimuli during a single experiment, such as baseline activity (application of KBS), mechanical distension, and chemical perfusion (vincristine, cinnamaldehyde, α,β-methylene ATP, and 6-shogaol, etc.). Between each stimulation, the tissue preparation was washed with fresh KBS buffer (10-mL for at least 10-min) until baseline fluorescence intensity stabilized.

**Figure 1:**
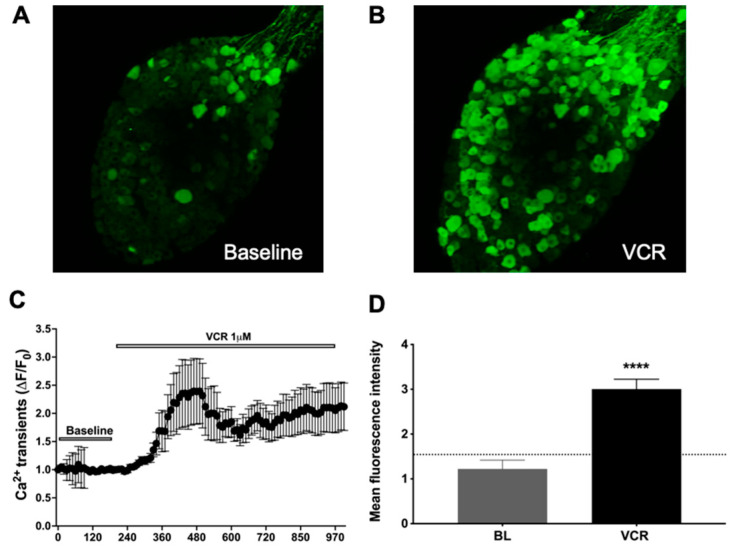
Vincristine-induced effect on vagal afferent neurons at their gastroesophageal nerve terminals. (**A**) Z stacked image of nodose ganglia at baseline; (**B**) Z stacked image of nodose ganglia during application of vincristine (VCR, 1 μM), showing responsive neurons (bright green). Responsive neurons were bright green due to VCR-evoked calcium transients. VCR was only applied to the gastroesophageal vagal afferent nerve terminals; (**C**) Representative traces of calcium transients (ΔF/F0) in nodose neurons that evoked by VCR; (**D**) Bar graph showing mean fluorescence intensity values for baseline (BL with KBS buffer: 1.06 ± 0.26 ) and VCR (3.8 ± 0.28) (BL vs. VCR, **** *p* < 0.0001, *n* = 257 neurons from 3 mice).

Application of vincristine (1 μM) at the vagal afferent nerve terminals in the esophagus and stomach for 2 min induced robust activation responses (as determined by calcium transients at the cell bodies), compared to baseline in nodose neuron cell soma in the ganglion. The mean intensity of vincristine response, at a concentration of 1 μM, was 3.8 ± 0.28 (*n* = 257 neurons from three mice) (Figure 1). We also found that vincristine, when applied directly on the ganglia without intact gastroesophageal tissue (no afferent nerves attached), was also able to cause a robust increase in calcium transients in nodose ganglia neurons (fluorescence intensity: 6.366 ± 0.34, *n* = 361 neurons from four mice).

### 2.2. Vincristine-Responsive Neurons Were Mainly Nodose C-Fiber Neurons

**Figure 2:**
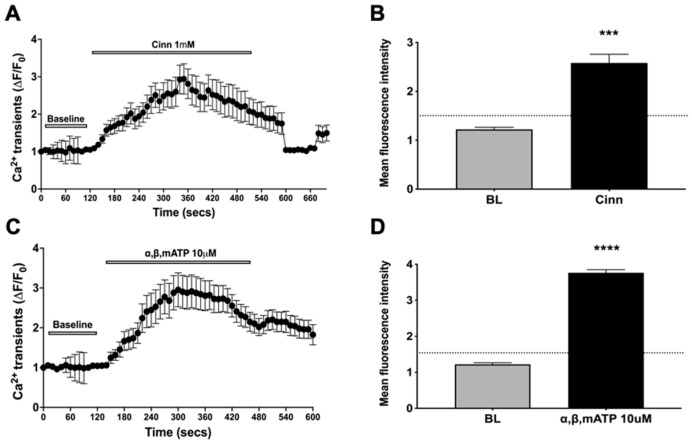
Characterization of vincristine-responsive gastroesophageal vagal afferent neurons. (**A**) Representative traces of calcium transients (ΔF/F0) evoked by cinnamaldehyde (cinn: 1 mM, 10 min) in 20 responsive nodose neurons; (**B**) Bar graph showing mean fluorescence intensity values for vehicle (KBS buffer, BL) and cinn (1.02 ± 0.09 vs. 2.58 ± 0.17, *** *p* < 0.001, *n* = 123 VCR-responsive neurons from three mice); (**C**) Representative traces of calcium transients (ΔF/F0) evoked by α,β-methylene ATP (α,β,mATP: 10 μM, 10 min) in 20 responsive nodose neurons; (**D**) Bar graph showing mean fluorescence intensity values for vehicle (KBS buffer, BL) and α,β,mATP (1.006 ± 0.08 vs. 3.764 ± 0.08, **** *p* < 0.0001, *n* = 192 VCR-responsive neurons from three mice).

We tracked the vincristine responsive neurons (vincristine applied onto the esophagus and stomach) for 4 min and washed the gastroesophageal-vagal preparations with fresh KBS for 30-min, then we applied the other C-fiber activators as we have previously characterized, including P2X_2/3_ agonist α,β-methylene ATP and TRPA1 agonist cinnamaldehyde [5,13]. Perfusion with α,β-methylene ATP significantly induced activation responses in more than half of vincristine-responsive neurons (75%) (total 257 neurons from three mice, paired experiments). The mean fluorescence intensities of activation increased from the baseline of 1.006 ± 0.08 to 3.764 ± 0.08, suggesting an activation effect to α,β-methylene ATP (Figure 2). Our previous studies have shown that P2X_2/3_ agonist α,β-methylene ATP selectively activated nodose but not jugular C-fiber neurons [5]. Thus, the high response rate to α,β-methylene ATP suggested their placode nodose neuron lineages. To confirm the responsive neurons were C-fiber neurons, we added TRPA1 agonist cinnamaldehyde, a stimulus selective for C-fiber nerves in the tissue. Perfusion with cinnamaldehyde significantly evoked activation responses in nearly half of vincristine-responsive neurons (48%) (total 123 out of 257 total VCR responsive neurons from three mice, paired experiments). The mean fluorescence intensities of activation increased from the baseline of 1.02 ± 0.09 to 2.581 ± 0.17, indicating that vincristine-activated nodose neurons were mainly C-fiber neurons. Similarly, perfusion with capsaicin, another C-fiber selective stimulus, also significantly evoked activation responses, mean fluorescence intensities from baseline 1.2 ± 0.1 to 2.884 ± 0.15, in nearly half of vincristine-responsive neurons (52%, 133 neurons out of 257 total VCR responsive neurons). Together, these data supported our hypothesis that vincristine directly activated vagal nodose C-fiber neurons at their gastroesophageal nerve terminals, and TRPA1 may play an import role in mediating such activation responses (Figure 2).

### 2.3. 5-HT3 Receptor Antagonist Significantly Blocked Vincristine-Induced Activation Response

**Figure 3:**
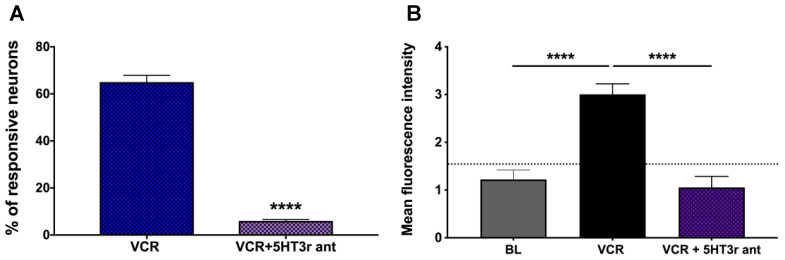
5-HT3 receptor antagonist blocked vincristine-induced activation responses in gastroesophageal vagal afferent neurons. (**A**) The percentage of responsive neuron in the vincristine (VCR, 1 μM, 30 min) group was 65 ± 5% (167 out of 257 recorded neurons from three mice). In the group (VCR + 5HT3r ant) that was pretreated with palonosetron (10 μM) for 30 min and then vincristine (1 μM) was applied in the presence of palonosetron for 30 min, the percentage of vincristine responsive neurons went down to 6 ± 1% (19 out of 329 recorded neurons from three mice) (VCR vs. VCR + 5HT3r ant, **** *p* < 0.0001); (**B**) The mean fluorescence intensity in the VCR group was 3.005 ± 0.2195, which significantly went down to 1.056 ± 0.2043 in the palonosetron treatment (VCR + 5HT3r ant) group (VCR vs. VCR + 5HT3r ant, **** *p* < 0.0001).

Palonosetron, a potent and selective 5-HT3 receptor antagonist, is a currently available anti-emetic drug for chemotherapy-induced nausea and vomiting. Our previous study demonstrated that 5-HT selectively activated nodose, but not jugular C-fibers, and such activation effect was mediated via 5-HT3 receptor [17]. Our present data demonstrated that pretreatment with palonosetron significantly inhibited vincristine-induced activation of gastroesophageal nodose C-fibers. Compared to vincristine (1 μM)-elicited activation responses in 65 ± 5% of the studied neurons (167 neurons were vincristine responsive out of a total of 257 recorded neurons from three mice), we observed that the percentage of vincristine responsive neurons went down to 6 ± 1% (19 neurons were vincristine responsive out of a total of 329 recorded neurons from three mice) when the preparations were pretreated with palonosetron (10 μM) for 30 min, and then vincristine (1 μM) in the presence of palonosetron (10 μM) was applied for 30 min. Similarly, the mean fluorescence intensity in the VCR group was 3.005 ± 0.2195, which significantly went down to 1.056 ± 0.2043 in the palonosetron treatment (VCR + 5HT3r ant) group (VCR vs. VCR + 5HT3r ant, **** *p* < 0.0001) (Figure 3).

### 2.4. TRPA1 Antagonist Significantly Inhibited Vincristine-Induced Activation Response

HC-030031 is a selective TRPA1 blocker, which has been shown to antagonize TRPA1 activation-elicited calcium influx without inhibition of TRPV1 functions [18]. To further address the role of TRPA1 in mediating the vincristine-induced activation response, we applied HC-030031 to determine whether it blocked the vincristine-induced effect.

**Figure 4:**
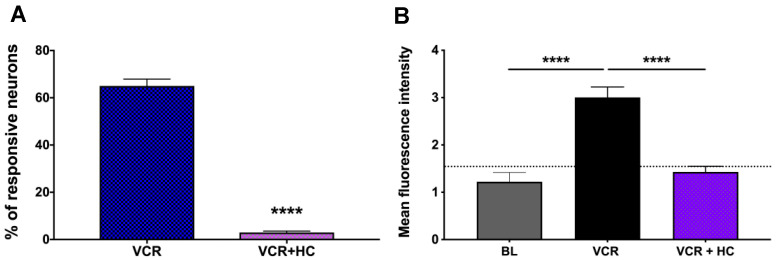
TRPA1 selective blocker HC-030031 significantly inhibited vincristine-induced activation responses in gastroesophageal vagal afferent neurons. (**A**) The percentage of responsive neuron in the vincristine (VCR, 1 μM, 30 min) group was 65 ± 5% (167 out of 257 recorded neurons from three mice). In the group (VCR + HC) that was pretreated with HC-030031 (100 μM) for 30 min and then vincristine (1 μM) in the presence of HC-030031 was applied for 30 min, the percentage of vincristine responsive neurons went down to 3 ± 1% (six out of 206 recorded neurons from three mice)(VCR vs. VCR + HC, **** *p* < 0.0001); (**B**) The mean fluorescence intensity in the VCR group was 3.005 ± 0.2195, which significantly went down to 1.429 ± 0.1189 in the HC-030031 treatment (VCR + HC) group (VCR vs. VCR + HC, **** *p* < 0.0001).

Compared to vincristine (1 μM)-elicited activation responses in 65 ± 5% of the studied neurons (167 vincristine neurons from a total of 257 recorded neurons from three mice), we observed that the percentage of vincristine responsive neurons went down to 3 ± 1% (six vincristine responsive neurons out of a total of 206 recorded neurons from three mice) when the preparations were pretreated with HC-030031 (100 μM) for 30 min, and then vincristine (1 μM) in the presence of HC-030031 was applied. Similarly, the mean fluorescence intensity in VCR group was 3.005 ± 0.2195, which significantly went down to 1.429 ± 0.1189 in the HC-030031 treatment (VCR + HC) group (VCR vs. VCR + HC, **** *p* < 0.0001) (Figure 4).

### 2.5. 6-Shogaol Significantly Attenuated Vincristine-Induced Activation Response

**Figure 5:**
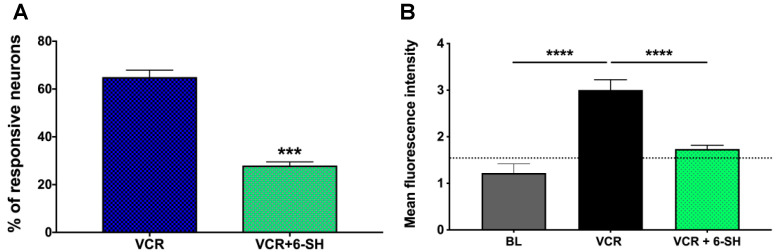
Ginger constituent 6-shogaol significantly attenuated vincristine-induced activation responses in gastroesophageal vagal afferent neurons. (**A**) The percentage of responsive neuron in the vincristine (VCR, 1 μM, 30 min) group was 65 ± 5% (167 out of 257 recorded neurons from three mice). In the group (VCR + 6-shogaol) that was pretreated with 6-shogaol (100 μM) for 30 min, and then vincristine (1 μM) in the presence of 6-shogaol (100 μM) was applied for 30 min, the percentage of vincristine responsive neurons significantly went down to 28 ± 3% (92 out of 329 recorded neurons from three mice)(VCR vs. VCR + 6-shogaol, *** *p* < 0.001); (**B**) The mean fluorescence intensity in the VCR group was 3.005 ± 0.2195, which significantly went down to 1.737 ± 0.0786 in the 6-shogaol treatment (VCR + 6-SH) group (VCR vs. VCR + 6-SH, **** *p* < 0.0001).

Ginger constituents have been used as a complementary medicine to relieve nausea and vomiting for a long time, but their neuronal action on emesis pathways is still unclear. Our recent study has demonstrated that 6-shogaol can not only activate but also desensitize gastroesophageal vagal nodose C-fibers via interaction with TRPA1 channels [13]. In the present study, 6-shogaol significantly attenuated vincristine-induced activation of gastroesophageal nodose C-fibers. Compared to vincristine (1 μM, 30 min)-elicited activation responses in 65 ± 5% of the studied neurons (167 vincristine responsive neurons out a total of 257 recorded neurons from three mice), we observed that the percentage of vincristine responsive neurons went down to 28 ± 3% (92 vincristine responsive neurons from a total of 329 recorded neurons from three mice) when the preparations were pretreated with 6-shogaol (100 μM) for 30 min, and then vincristine (1 μM) in the presence of 6-shogaol (100 μM) was applied for 30 min. Similarly, the mean fluorescence intensity in the VCR group was 3.005 ± 0.2195, which significantly went down to 1.737 ± 0.0786 in the 6-shogaol treatment (VCR + 6-SH) group (VCR vs. VCR + 6-SH, **** *p* < 0.0001) (Figure 5).

## 3. Materials and Methods

### 3.1. Animals

Male C57BL/6J mice (4–6 weeks) were purchased from the Jackson Laboratory. Pirt-GCaMP6s mice were bred by crossing Pirt-Cre with Rosa26-LoxP-STOP-LoxP-GCaMP6s mice at the animal care facility, Johns Hopkins University School of Medicine. Pirt-GCaMP6s heterozygotes were used in two-photon neuron imaging experiments. The total number of the animals used in the study was 16. All animals were kept in pathogen-free conditions and fed with standard food and clean water. All experiments were approved by the Johns Hopkins University Animal Care and Use Committee (approval protocol number: MO19M121). When harvesting tissues for ex vivo gastroesophageal-vagal preparation, the animals were put into a chamber and euthanized by inhalation of compressed CO_2_ gas from a cylinder.

### 3.2. Chemicals

The chemicals used in the experiments were all purchased from Millipore Sigma (St. Louis, MO, USA), including vincristine sulfate, cinnamaldehyde, α,β-methylene ATP, 6-shogaol, TRPA1 antagonist HC-030031, and 5-HT3 receptor antagonist palonosetron hydrochloride. Palonosetron was prepared with DMSO as stock solution and then diluted with KBS when applied. All other chemicals (vincristine, cinnamaldehyde, α,β-methylene ATP, 6-shogaol, HC-030031) were prepared freshly with KBS within one hour before application.

### 3.3. Two-Photon Nodose Neuron Imaging Using Pirt-GCaMP6s Mouse

Pirt-Cre;R26-GCaMP6s transgenic mice were used for two-photon imaging studies according to our previously described method [13]. We developed the gastroesophageal-vagal preparations with intact nerve endings in either the esophagus or the stomach, based on the methods as described in our previous studies [13]. After the animals were killed by CO_2_ inhalation and exsanguination, the esophagus and stomach, with intact bilateral extrinsic vagal nodose ganglia, were dissected and pinned in a Sylgard-lined Perspex chamber with tissue and ganglia compartments, which were separated using Vaseline and superfused with Krebs bicarbonate buffer (KBS, pH 7.4, 35 °C, 4–6 mL/min) without fluid exchange (KBS, composed of (mM): NaCl, 118; KCl, 5.4; NaH2PO4, 1.0; MgSO_4_, 1.2; CaCl_2_, 1.9; NaHCO_3_, 25.0; and dextrose, 11.1, and gassed with 95% O_2_–5% CO_2_, pH7.4, 35 °C). Polyethylene tubing was inserted 3–5 mm into the cranial esophagus and the antrum and then secured for distension. The left or right vagal ganglia were pinned in a separate compartment for two-photon imaging. The chamber was mounted on the microscope stage and fixed in place with 2 screws. Both tissue and ganglion compartments were then connected independently to buffer inlet lines perfusing warm buffer (35 °C), heated via Warner instruments heating elements. The flow of KBS, gassed with air mix (95%O_2_–5%CO_2_, pH 7.4), was maintained at 4-mL per min. The 20× water immersion objective (Olympus, Center Valley, PA, USA) was positioned directly above the ganglion compartment. Using LabVIEW software (National Instruments, Austin, TX, USA), the ganglia coordinates for Z stack, starting from the top of the ganglion to the bottom (~100 microns), were adjusted. The piezo drive (Scientifica, Uckfield, UK) for the objective was engaged, and the objective was positioned at the top of the ganglia for image acquisition. We acquired live images of the nodose ganglia at 10 frames with 600 Hz frame scan mode per 6 s at a depth of ~100 μm (10 planes of 10 μm thick slices). In order to calibrate the two-photon system for our ex vivo preparation, we carried out extensive electrical stimulation of the vagal nerve trunk and analyzed the Ca^2+^ transients to show that the intensity of the response was quasi-linear between 1 Hz and 10 Hz stimulations (when given in 5 s trains, data not shown). First, we recorded baseline activity of the neurons without any stimulation and adjusted the laser power and emission gain (of the PMT) to record the lowest signal to noise ratio. The laser power and gain for our system were adjusted at 35% and 600 volts, respectively. Next, we delivered 1 mL of KBS into the lumen of gastroesophageal preparation and recorded activity of neurons that positively responded to such intra-lumen distention. We marked these neurons during data analysis. The two-photon setup used a laser excitation wavelength of 920 nm, which prevented photobleaching of the GCaMP6, and hence, there was no loss of signal over repetitive stimulation. The drugs were perfused to the serosal surface of the stomach and the esophagus, and the responses were recorded for a total of 3–6 min. The gastroesophageal preparation was washed with 10 mL of KBS in between every drug application. The baseline was recorded for 10 s before each drug application. A neuron in the vagal ganglia is considered responsive when it responds to any of the following stimuli during the experiment, including both mechanical (distention) and chemical (drug application with VCR, Cinn, abmATP, etc.) stimuli. The neuron is considered responsive when its mean fluorescence intensity in a given stimulation goes higher than 1.5 times over the baseline intensity. Baseline intensity usually is approximately 1 ± 0.2, as some neurons have spontaneous activities. The recorded images were saved in tiff files and analyzed offline using ImageJ (NIH, Bethesda, MD, USA). Data were presented as ΔF/F0, the change in fluorescence intensity from baseline.

### 3.4. Data Analysis

In two-photon imaging, we usually imaged the nodose ganglion up to a depth of 100 microns in increments of 10 microns (1 plane). So, altogether, 10 planes were imaged in 6 secs (per cycle). Typically, 1 cycle of images was collected as baseline (F0) before any drug was applied. Images were collected for 3–6 min during drug application. All images were exported as tiff files to ImageJ for offline analysis. The first step in ImageJ was to open the Z-stacks of the various applications individually and observe the time lapse images using group Z-stack compression. This helped to determine if there was any X, Y, or Z axis movement of the ganglia during recording. The movement of the ganglia during recording was corrected using an ImageJ plugin Stackreg. Next, the Z-stacks for multiple drug analysis were concatenated (stitched) together in the sequence they were applied. The concatenated images were then divided or separated into substacks (each Z stack was divided by 10 to create a substack), which were separated by 20 microns. The 20-micron separation was chosen as it gave enough separation between stacked neurons in the ganglia (mouse sensory neurons are approximately 20-micron in diameter). Thus, 5 substacks out of the 10 were analyzed, which ensured the same neurons were not counted twice. The substacked images were used to mark the responsive neurons, and the intensity values (ROIs) were collected and then analyzed further in Excel to calculate the ΔF/F0 value (change in fluorescent intensity from baseline). Mean fluorescence intensity values were calculated by averaging the maximum ΔF/F0 values in the presence of a drug application. The data were presented as mean ± SEM and compared by unpaired t-tests. For all experiments, significance was defined as *p* < 0.05.

## 4. Discussion and Conclusions

Ginger has a long history of being used as a complementary medicine to relieve nausea and vomiting, but its neuronal action on emesis pathways is still unclear. After ingestion, ginger and its metabolites are highly accumulated in the GI tract; this enables them to exert considerable influence on vagal afferent nerve endings in the proximity. Two major ginger constituents, 6-gingerol (fresh ginger) and 6-shogaol (dry ginger), have been used to alleviate GI symptoms such as postoperative and chemotherapy-induced nausea and vomiting [7,8]. The present study aimed to determine whether ginger constituent 6-shogaol can prevent chemotherapy agent vincristine-elicited effects on gastroesophageal vagal afferent nerve terminals.

Our data demonstrate that vincristine activates a large percent of vagal nodose nociceptive C-fiber neurons at their nerve terminals in the gastroesophageal tissue. This activation is largely inhibited by the 5-HT3 receptor antagonist palonosetron, a drug effective in inhibiting vincristine-induced emesis. This supports the hypothesis that at least part of the mechanism of this compound is due to inhibitory effects on vagal C-fiber terminals in the gut tissue. Similarly, the data also support the hypothesis that ginger constituents such as 6-shogaol owe part of their anti-nausea/emetic effects from an inhibition of vagal C-fibers’ activation at nerve terminals.

As a chemotherapy agent, vincristine can disrupt microtubule aggregation and lead to mitotic arrest and cell death [19]. Such toxic actions are not limited to cancer cells, but may extend to many other cells, including sensory neurons. Vincristine-elicited neuronal actions have been mainly investigated in chemotherapy-induced peripheral neuropathy (CIPN) in both animal models and human subjects [20]. However, evidence of their direct action at afferent nerve endings is still missing. The present study added new knowledge and demonstrated that vincristine directly activated vagal nociceptive afferent C-fiber neurons at their gastroesophageal nerve terminals. Such nerve ending effect seems unlikely to be secondary to the release of mediators in the tissue, as we noted that vincristine can directly activate the cell bodies in the vagal sensory ganglion (devoid of gastroesophageal tissue).

The observation that most, but not all, of the nerve fibers responding to vincristine could also be activated by C-fiber selective stimuli, such as the TRPV1 agonist capsaicin and the TRPA1 agonist cinnamaldehyde, indicates that most vincristine-activated vagal afferent nerves are C-fibers. Whether the vincristine-sensitive nerves, but insensitive to capsaicin or cinnamaldehyde, were Aδ-fibers or TRPV1/TRPA1 negative C-fibers is unknown.

Vagal afferent C-fibers innervating visceral tissues are derived from neurons situated in either the nodose or jugular vagal ganglia. In the mouse, these two disparate ganglia are often fused into a vagal ganglion complex [21]. We have previously noted that P2X_3_/P2X_2/3_ receptor agonist α,β-methylene ATP selectively activates nodose afferent nerve terminals, but not jugular nerve terminals [5]. This activation is secondary to stimulation of P2X_2/3_ receptors. Our present data also shows that most vincristine-activated neurons were also responsive to α,β-methylene ATP, indicating they were nodose C-fibers. Those that did not respond to α,β-methylene ATP might be P2X_2/3_ negative nodose nerve fibers or jugular C-fibers.

The molecular mechanism by which vincristine activated vagal C-fibers in the gastroesophageal tissue defies a simple explanation. The blocking of 5-HT3 receptors that nearly abolished the response indicates that serotonin is involved in the activating mechanism. Serotonin is an abundant mediator that is stored in enterochromaffin (EC) cells in the GI tract. It would therefore appear that vincristine evokes the release of serotonin from EC cells, then stimulates the ionotropic 5-HT3 receptors on C-fiber terminals leading to action potential discharge. Similar action mechanism has also been implicated in vincristine-induced neuropathy [22].

The data also reveals that blocking TRPA1 channels strongly inhibits the C-fiber activation by vincristine. This suggests that vincristine may be capable of directly activating TRPA1 channels on the C-fibers. This would explain how vincristine directly activated the neuronal cell bodies in the ganglia, independent of enterochromaffin cells. That blocking either 5-HT3 receptors or TRPA1 channels alone had a strong inhibitory effect could be explained by the hypothesis that vincristine-induced action potential discharge requires a depolarization (generator potential) caused by cations crossing the terminal membrane through both TRPA1 channel and 5-HT3 receptor. Another possible explanation for this observation is that vincristine-induced release of 5-HT from EC cells requires activation of TRPA1 on the EC cells [23]. It is interesting to note that both 5-HT3 and TRPA1 have also been deemed causal in vincristine-induced peripheral neuropathies [22,24]. Previous studies have also revealed an important role of TRPA1 in chemotherapy agent-elicited functional responses in DRG neurons [25].

Our recent study demonstrated that dry ginger constituent 6-shogaol evokes robust action potential discharges and calcium influxes in vagal nodose C-fiber neuron nerve terminals via activation of TRPA1. After the response terminated, the fibers are desensitized to further stimulation via TRPA1 mechanisms [13]. Our present data showing that 6-shogaol significantly attenuated vincristine-induced activation response in gastroesophageal nodose C-fibers is therefore likely explained by a desensitization of TRPA1 channels.

In conclusion, the present study provides new evidence that chemotherapeutic agent vincristine strongly activates vagal afferent nociceptive nodose C-fibers at their gastroesophageal nerve endings. The full activation of the C-fibers by vincristine requires both 5-HT3 receptors and TRPA1 channels. This knowledge may help to better understand the mechanism of vagal afferent nerve-mediated emetic reflex and may lead to the development of new and alternative approaches to alleviate chemotherapeutic agent-induced adverse emetic effects.

## Data Availability

Not applicable.

## Abbreviations

Pirt: phosphoinositide interacting regulator of transient receptor potential channels; TRPA1: transient receptor potential cation channel subfamily A member 1; GCaMP6: GCaMP6 belongs to a family of ultrasensitive fluorescent calcium sensors, consisting of circularly permuted GFP (cpGFP), the calcium-binding protein calmodulin (CaM), and CaM-interacting M13 peptide; P2X_2/3_: P2X purinoceptor _2/3_.
